# Halitosis in young patients with chronic kidney disease: findings from a randomized controlled trial

**DOI:** 10.1186/s13005-024-00428-y

**Published:** 2024-05-15

**Authors:** Karolin Charlotte Hoefer, Anna Greta Barbe, Anne Adams, Christoph Schoppmeier, Michael Jochen Wicht, Lutz T Weber, Michael J Noack, Isabelle Graf

**Affiliations:** 1 Department of Operative Dentistry and Periodontology, Faculty of Medicine, University Hospital of Cologne, University of Cologne, Cologne, Germany; 2 Institute of Medical Statistics and Computational Biology, Medical Faculty, University of Cologne, Cologne, Germany; 3 Department of Pediatric Nephrology, Children’s and Adolescents’ Hospital, Faculty of Medicine, University Hospital of Cologne, University of Cologne, Cologne, Germany; 4 Department of Orthodontics, Faculty of Medicine, University Hospital of Cologne, University of Cologne, Cologne, Germany

**Keywords:** Chronic kidney disease, Halitosis, Children, Adolescents

## Abstract

**Background:**

Chronic kidney disease (CKD) directly affects oral health. Yet data about halitosis in young CKD patients and the impact of dental prophylaxis is limited. Therefore, as part of this randomized clinical trial, halitosis in young CKD patients undergoing intensive or standard oral preventive procedures was to be explored.

**Methods:**

Three volatile sulfur compounds (hydrogen sulfide, methyl mercaptan and dimethyl sulfide) were measured in 30 young patients with CKD (mean age 14.2 years; 16 males, 14 females). Breath samples were taken after 3 and 6 months and analyzed with selective gas chromatography (OralChroma). Tongue coating (Winkel Index) and clinical indices to determine local inflammation or oral hygiene (Papillary Bleeding Index and Quigley-Hein Index) were assessed. Within an extended anamnesis, patients and their mothers and nurses were questioned about the perceived halitosis. Corresponding quotes were noted verbatim. Patients were randomized to either intensive need-related oral health care measures (oral preventative program, OPP) or a one-stage standard prevention (treatment as usual, TAU).

**Results:**

While there were no differences in volatile sulfur compound levels between TAU and OPP at the three time points of measurements (*p* > 0.05), there was a tendency towards a reduction in dimethyl sulfide and hydrogen sulfide of affected patients within the OPP group over time. Looking at potential differences between both groups with regard to tongue coating, significant differences were observed between baseline and 3 months after study start in the OPP group, and between baseline and 6 months after study start in the TAU group (*p* < 0.05). The burden of halitosis was frequently reported by patients’ mothers and nurses.

**Conclusions:**

Young CKD patients regularly suffered from halitosis and dimethyl sulfide was its main source. Preventive measures mainly resulted in a reduction of tongue coating. Trial registration: The German Clinical Trial Register (# DRKS00010580).

## Background

Halitosis generally affects a large proportion of humans, either in the short or long term. The reported prevalence of halitosis varies greatly and lies between 8% and 45% in young patients [1–3]. The inconsistency could be due to the lack of standardization in halitosis detection and threshold criteria [4]. Only limited data is available about the prevalence of halitosis in children and adolescents [5], but there are reports that halitosis appears to be particularly challenging in adult patients with chronic kidney disease (CKD) [5].

In general, oral malodor is caused by a high local concentration of intraoral microbial populations, particularly those on the tongue biofilm, as well as the biofilms associated with teeth and periodontal tissue [6]. Halitosis is linked to poor oral hygiene, dental plaque, caries, gingivitis, stomatitis, periodontitis, tongue coating and oral carcinoma. Up to 90% of all cases of bad breath are due to disorders in the oral cavity caused by anaerobes [7]. The sulfur-containing amino acids cystine, cysteine and methionine are degraded to the foul-smelling volatile sulfur compounds (VSC) hydrogen sulfide (HS) and methyl mercaptan (MM). While dimethyl sulfide (DMS) seems to be only a minor component of oral malodor, it is caused by conditions other than those already mentioned [7]. Among the odor-intensive intraoral volatile substances, extra-oral odorous volatiles can be absorbed into the bloodstream from any source in the body (e.g. mouth, stomach, intestine and liver) and later transmitted to the alveoli [8]. Pulmonary excretion of these volatiles into the respiratory air then results in halitosis when the malodorous volatiles are present in objectionable concentrations in the breath [7]. A so-called extra-oral blood-borne halitosis, comprising 5–10% of all cases of bad breath, can generally be caused by some systemic diseases, metabolic disorders (such as hepatic failure, liver cirrhosis uremia, diabetic ketoacidosis, diabetes mellitus), medication intake or food consumption (e.g. garlic or onion) [2, 9]. Individuals with halitosis are often burdened by psychosocial impairments [10, 11] and consequently a low oral health-related quality of life [12].

In patients with advanced CKD, an ‘uremic fetor’ can frequently be detected, which is mainly attributed to the exhalation of ammonia, dimethylamine and trimethylamine. In this context, intraoral conditions such as gingivitis are also regularly observed. Although many cross-sectional studies have been performed to demonstrate that higher VSC values are present before gingivitis treatment than after, randomized clinical trials are needed [13]. Due to limited data in young patients [14], especially in diseased children and adolescents, it remains questionable whether elevated concentrations of HS, MM and DMS are detectable and whether these gases can be reduced by the treatment and need-based prophylaxis of gingivitis. Furthermore, the clinical significance of possible halitosis has to be determined in terms of self- and proxy-reported potential impairments due to noticeable bad breath.

In this secondary analysis of a longitudinal study, in which a need-based prophylaxis program for gingivitis management in young CKD patients was applied, the aim was to determine whether increased VSC values were present in young CKD patients. In addition, associations between gingivitis, gingivitis-relevant indices, tongue coating and halitosis VSC values were investigated. The influence of a prophylaxis program on VSC scores was longitudinally evaluated, with a particular focus on the Winkel Index after tongue cleaning [15].

Thus, the purpose of this part of this randomized clinical trial was to evaluate the existence of halitosis in young CKD patients and determine the significance of individual VSC components before and after an oral health prevention program.

## Methods

### Study design

This secondary analysis was part of a clinical trial which was performed as a prospective, single-center, randomized controlled clinical trial (see CONSORT checklist). The effects of an intensive oral prophylaxis program (= OPP) were compared with standard statutory health insurance prophylaxis (treatment as usual = TAU) on halitosis among young patients with renal insufficiency. OPP included instructions related to tongue cleaning, among others. The trial was approved by the Ethics Committee of the Faculty of Medicine at the University Hospital of Cologne (#15–264) and recorded at The German Clinical Trials Register (#DRKS00010580).

### Patient recruitment and randomization

**Fig. 1:**
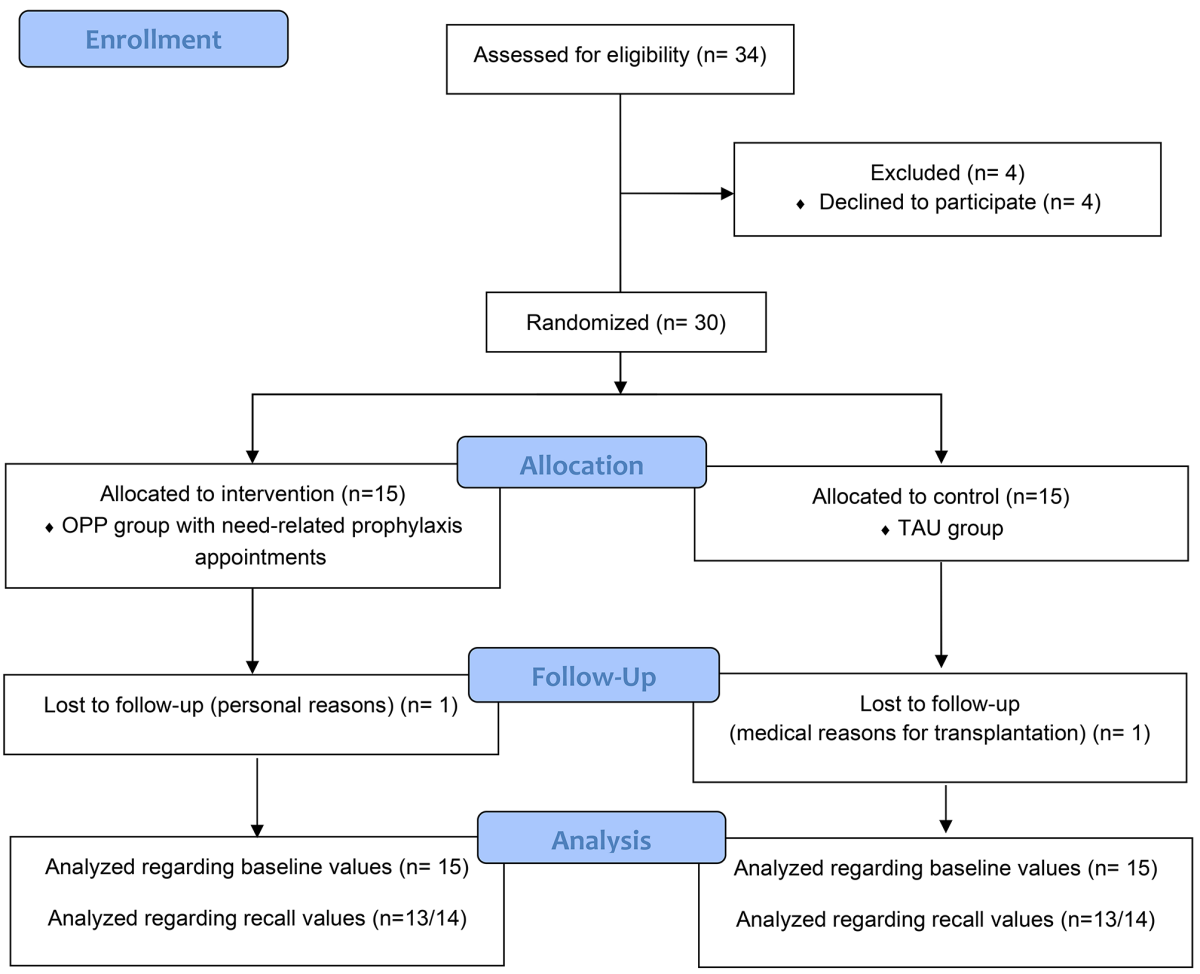
CONSORT flow chart

**Fig. 2:**
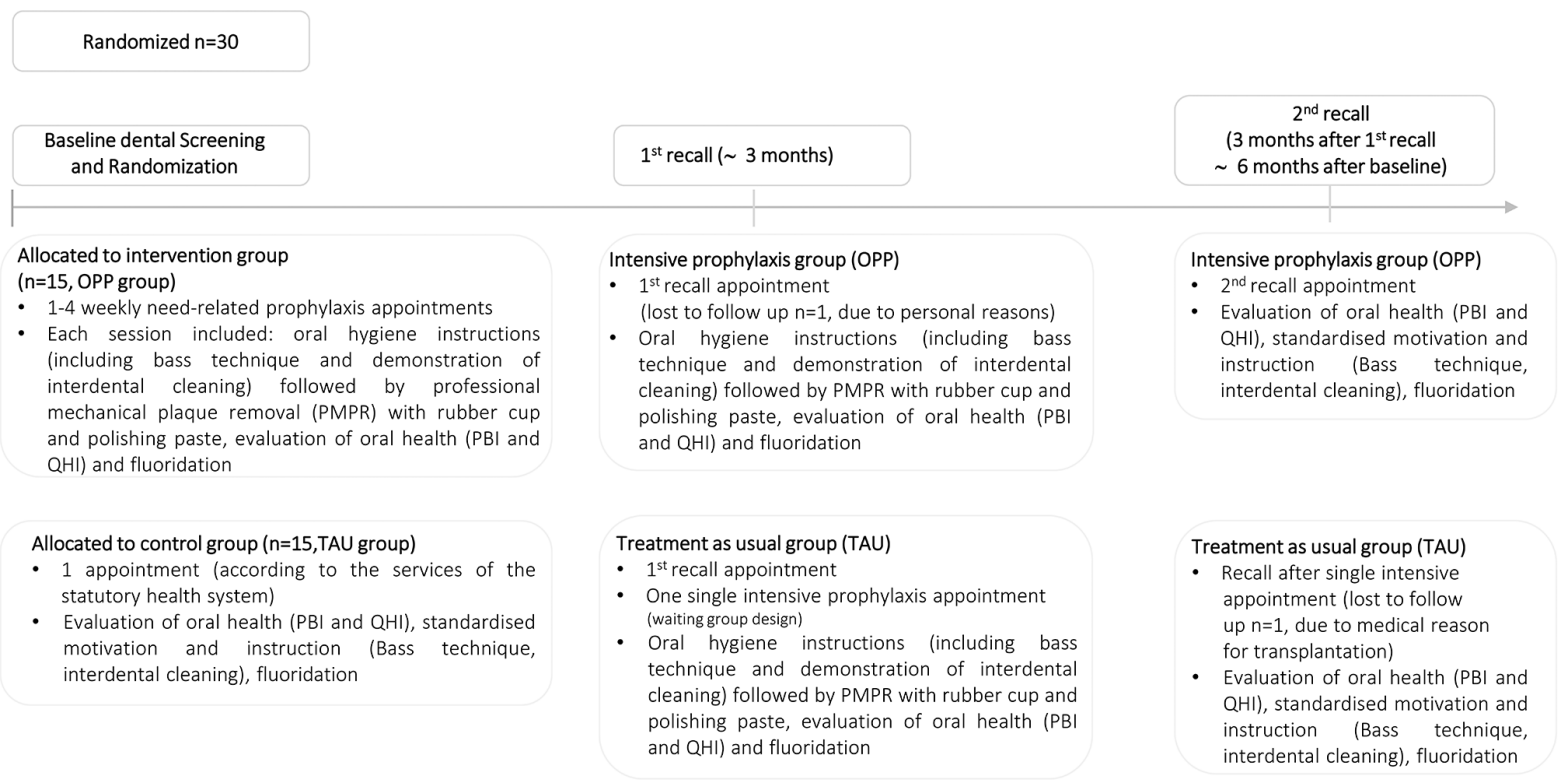
Timeline of the study

Patients were initially screened for study enrollment by pediatric nephrologists and then pediatric dentists, and randomly assigned to the intervention or control group via a computer-guided program, TENALEA (ALEA, Abcoude, NL). Patients attending the Department of Pediatric Nephrology at the University Hospital who fulfilled the following inclusion criteria were enrolled in the study: patients with CKD grade 1–5 according to the ‘Kidney Disease: Improving Global Outcomes’- (KDIGO)- classification [criteria for advanced CKD according to the glomerular filtration rate and albuminuria [16]], patients undergoing conservative treatment, and those already transplanted or dialyzed; patients with gingivitis. Any signs of acute infection and/or fever or antibiotic treatment in the 14 days prior to participation were defined as exclusion criteria. This decision was made by the treating pediatric nephrologists based on clinical parameters and blood tests. Figure 1 shows the CONSORT flow chart and Fig. 2 highlights the timeline of this study.

### Study measures

The main parameters studied were the VSCs HS, DMS and MM, measured by gas chromatography (OralChroma™ CHM-1) and the Winkel Tongue Coating Index [15] as well as the Quigley Hein Index (QHI) and the Papillary Bleeding Index (PBI) at three time points: baseline, 3 months after dental prophylaxis, 6 months after baseline. At baseline, an intraoral dental examination, including dmft/DMFT caries diagnostics (according to the International Caries Detection and Assessment System) was performed. Caries restorative measures were carried out when needed. The focus of this paper lies on the halitosis-related measurements such as VSCs and Winkel Index. OralChroma uses an indium oxide semiconductor gas sensor; it does not require a carrier gas like conventional gas chromatographs, but instead uses room air as a carrier for the chromatography column. To ensure the validation of VSC measurements, the OralChroma device was calibrated by the manufacturers (Abilit Corporation, Japan) before the study began according to their guidelines. OralChroma has been described as one of the most appropriate methods to detect halitosis of different origins (intra- and extra-oral halitosis) [17]. Patients were instructed to refrain from foods containing garlic, onion and spices 24 h prior to measurements. Furthermore, tooth brushing, mouth rinsing or chewing gum were only permitted up to 1 h prior to measurements. All measurements were supposed to take place in the mornings and were carried out by the same examiners (KH and IG). For sample collection, a disposable syringe (0.5 mL) was inserted two-thirds into the patient’s oral cavity for 30 s. The plunger was slowly pulled out and pushed back in twice more, before the definitive sample was taken and injected into the analyzer. After processing was completed, the concentration of VSC was visualized in parts per billion (ppb) by the OralChroma Data Manager software package. As the cognitive threshold values are reported differently in the literature, relevant limits of detection were chosen as 95 ppb for HS, 12 ppb for MM and 24 ppb for DMS, in accordance with Tangermann und Winkel [18].

In addition, the Tongue Coating Index according to Winkel was measured. For this Winkel Index, the tongue was imaginarily divided in six areas- three anterior and three posterior parts. Tongue coating was then scored as 0 = no coating, 1 = light coating or 2 = severe coating; in general, a score between 0 and 12 is the result [13].

Furthermore, short, narrative interviews were conducted with all patients and their mothers mainly regarding self-perceived oral malodor in the course of an extended anamnesis in order to determine potential impairments due to possible halitosis. In addition, five nurses were asked about their perceptions of halitosis in young CKD patients during study-related visits at the CKD unit. Quotes from these discussions were recorded verbatim. The qualitative content analysis was performed by two experienced researchers (KH and IG) according to Mayring [19] and revolved around the (perceived) presence of halitosis.

The dental examinations were conducted by two calibrated specialized pediatric dentists. Within the first part of the study, the test group (oral preventative program = OPP) received one to four need-based prophylaxis sessions until gingivitis improved (according to PBI, no bleeding) while the control group (treatment as usual = TAU) received one dental prophylaxis session that was adapted to the guidelines of the statutory health insurances with the means of motivational instruction in a professional dental setting. All patients received individualized oral hygiene instructions including the bass technique and a demonstration of interdental cleaning. 3 months after the study onset, all included patients were re-examined and the control group received a single, supplementary, single intensive prophylaxis session (including professional mechanical plaque removal). The final examination of all study patients took place 6 months after the trial had started (Fig. 2). For the secondary analysis focusing on halitosis, the test group (OPP) was regularly instructed to perform mechanical tongue cleaning, which was regularly examined using the Winkel index. The control group (TAU) also received the instruction for mechanical tongue cleaning after 3 months.

### Sample size calculation

The present study focused on halitosis as a secondary endpoint; the sample size was calculated for the primary endpoint gingivitis (PBI), using the statistical software package (G* Power, University Düsseldorf). The following a priori parameters were used: expected reduction in gingivitis [20] of 1 PBI value with a standard deviation (SD) 0.7 and non-normally distributed values (non-parametric tests), two-sided analysis, probability of error of 5% and a power of 80%, and a high dropout rate. The calculation resulted in a total of 24 patients, with an additional six assumed drop-outs, which resulted in a two-armed study with 15 patients per group.

### Statistical analyses

All statistical analyses were performed using IBM SPSS Statistics 28 (IBM Corp. Released 2021, IBM SPSS Statistics for Windows, Version 28.0. Armonk, NY: IBM Corp). Patient and treatment characteristics were compared by the Mann-Whitney U test for continuous variables and Pearson’s chi-square tests for categorical variables where appropriate. Differences in DMS, HS, MM and Winkel Index between the test and the control group at baseline, 3 and 6 months were analyzed using the Mann-Whitney U test. Wilcoxon rank-sum tests were performed to detect significant changes in DMS, HS, MM and Winkel Index within groups at different time points. Furthermore, correlation analyses were performed to identify correlations between the parameters of interest. Statistical significance was set at *p* < 0.05.

## Results

### Patients

30 patients were enrolled (*n* = 15 per group). The mean age was 13.5 years in the OPP group (10 males, five females) and 14.9 years in the TAU group (six males, nine females) (Table 1). No statistically significant differences were detected between females and males at any of the three time points with regard to VSC gases or tongue coating (*p* > 0.05). The most frequent chronic kidney diseases among the included patients were congenital anomalies of the kidney and urinary tract (CAKUT) (OPP group *n* = 8; TAU *n* = 3), glomerulopathies (OPP *n* = 2; TAU *n* = 7) and ciliopathies (OPP *n* = 3; TAU *n* = 4).

**Table 1:** Baseline demographic and clinical characteristics

	Total (*n* = 30)	OPP (*n* = 15)	TAU (*n* = 15)	*p*-value
Sex				0.143 ^*a*^
male	16 (53.3)	10 (66.7)	6 (40)	
female	14 (46.7)	5 (33.3)	9 (60)	
Age in years				0.539 ^*b*^
mean ± SD	14.2 ± 5.2	13.5 ± 4.9	14.9 ± 5.5	
range	6–26	6–21	7–26	
Dentition				0.456 ^*a*^
mixed	12 (40)	7 (46.7)	5 (33.3)	
permanent	18 (60)	8 (53.3)	10 (66.7)	
DMFT/dmft				0.935^b^
mean ± SD	0.6 ± 1.0	0.6 ± 1.0	0.7 ± 1.0	
PBI				
baseline	1.1 ± 0.7	1.0 ± 0.6	1.1 ± 0.8	0.935^b^
after 3 months	0.5 ± 0.7	0.1 ± 0.1	1.0 ± 0.7	
after 6 months	0.3 ± 0.2	0.2 ± 0.1	0.4 ± 0.3	
QHI				
baseline	2.5 ± 0.9	2.6 ± 1.0	2.3 ± 0.9	0.233^b^
after 3 months	1.4 ± 1.0	0.7 ± 0.5	2.1 ± 0.8	
after 6 months	1.0 ± 0.6	0.8 ± 0.6	1.2 ± 0.6	
Primary disease				0.085 ^*a*^
CAKUT	11 (36.7)	8 (53.3)	3 [20]	
Glomerulopathy	9 [30]	2 (13.3)	7 (46.7)	
Ciliopathy	7 (23.3)	3 [20]	4 (26.7)	
Systemic disease	1 (3.3)	0 (0)	1 (6.7)	
Renovascular	0 (0)	0 (0)	0 (0)	
Others	2 (6.7)	2 (13.3)	0 (0)	
Therapy				0.606 ^*a*^
Conservative	7 (23.3)	2 (13.3)	5 (33.3)	
Dialysis	3 [10]	2 (13.3)	1 (6.7)	
Post-transplant	20 (60)	11 (66.7)	9 (53.3)	
Medication				
Immuno-suppression	22 (73.3)	11 (73.3)	11 (73.3)	
Cyclosporin	5 (16.7)	3 [20]	2 (13.3)	
Amlodipine	17 (56.7)	8 (53.3)	9 (60)	
Ramipril	11 (36.7)	6 (40)	5 (33.4)	
Amlodipine + ramipril	4 (13.3)	2 (13.3)	2 (13.3)	

^*a*^ Pearson’s chi-squared test; ^*b*^ Mann–Whitney U test. CAKUT, congenital anomalies of the kidney and urinary tract; OPP, oral preventive program; PBI, papillary bleeding index; QHI, Quigley–Hein plaque index; TAU, treatment as usual. Characteristics for categorical variables are presented as n (%), continuous variables as mean ± standard deviation (SD)

### Volatile sulfur compounds

After analysis of all patient data, a mean HS level of 102.1 ppb (SD 303.9; range 0-1633.5; mean levels of 50.0 ppb in OPP and 154.1 ppb in TAU group) was observed for both groups at baseline. The HS level was 175.4 ppb (SD 676.0; range 0-3475.5) after 3 months and 62.6 ppb (SD 132.7; range 0-505.0) after 6 months.

At baseline, the mean MM level was 31.7 ppb (SD 106.5; range 0-581.5; mean levels of 17.1 ppb in OPP and 46.2 ppb in TAU group) within both groups at baseline, 22.6 ppb (SD 57.2; range 0-246.0) after 3 months and 22.3 ppb (SD 43.1; range 0-203.5) after 6 months.

For DMS, a mean of 47.3 ppb (SD 42.4; range 0-148.5; mean levels of 46.2 ppb in OPP and 48.4 ppb in TAU group) was observed at baseline. After 3 months and 6 months, the mean DMS levels were 74.6 ppb (SD 118.6; range 0-499.0) and 63.3 ppb (SD 134.9; range 0-679.0), respectively.

Table 2 shows all measured VSCs in both groups for the three relevant points of time.

**Table 2:** OralChroma™ VSC measurements at baseline, 3 and 6 months in young CKD patients

Patient ID / group	Dimethyl sulfide (ppb)			Hydrogen sulfide (ppb)			Methyl mercaptan (ppb)		
	Baseline	3 months	6 months	Baseline	3 months	6 months	Baseline	3 months	6 months
**OPP**									
9 (*)	0	**197**	**110**	0	0	4	0	0	**39**
19	0	0	0	0	31	2	0	0	0
28	12	m.v.	m.v.	**232**	m.v.	m.v.	**53**	m.v.	m.v.
10	21	m.v.	0	14	m.v.	0	0	m.v.	0
23	21	**342**	0	0	0	0	0	**165**	**18**
21	**40**	**73**	0	11	43	0	0	**43**	5
2	**48**	0	21	4	46	0	0	0	0
33	**48**	0	0	0	23	**266**	0	0	**204**
1	**49**	0	**36**	11	4	0	5	0	10
16	**49**	**499**	**28**	0	**138**	14	0	**246**	0
13	**61**	12	0	35	10	2	0	0	0
24	**64**	**96**	0	**446**	19	15	**95**	0	0
8	**69**	**149**	**83**	0	0	0	0	0	0
29(*)	**84**	0	12	0	0	0	**43**	0	0
32	**130**	12	0	0	0	61	**62**	0	**107**
**TAU**									
5	0	**24**	0	**158**	**101**	58	10	**24**	10
7	0	**73**	21	74	78	4	0	**18**	0
18	0	21	**42**	0	13	2	0	10	10
20	0	0	0	13	0	18	0	0	0
22	0	**36**	**115**	11	71	**356**	0	0	**58**
17	12	0	**33**	0	0	0	0	0	**19**
12	21	**84**	**120**	**111**	**264**	21	27	**65**	**31**
26	**42**	0	**100**	**133**	0	**347**	**31**	0	0
34	**42**	**178**	**679**	**1634**	**3476**	**505**	**582**	0	**43**
25	**64**	**40**	0	19	**129**	0	0	**18**	0
3	**77**	**28**	**42**	15	**105**	58	**41**	0	5
31	**95**	m.v.	**303**	35	m.v.	0	0	m.v.	**47**
11	**100**	m.v.	**95**	16	m.v.	23	5	m.v.	**23**
30	**127**	0	0	23	12	0	0	0	0
14	**149**	**79**	0	71	0	0	0	0	0

Measurements above threshold according to Tangermann and Winkel highlighted in **bold** * Chlorhexidine mouthwash after baseline within the TAU group; m.v. missing value

Table 3 shows the distribution of “affected” and “not affected” patients with regard to the previously described thresholds of clinically noticeable halitosis, grouped for the three VSCs of interest in the OPP and TAU groups. While no statistically significant differences in VSC levels were found between TAU and OPP at the three time points, there was a tendency towards a reduction in DMS and HS in affected patients within the OPP group over time (for DMS: 67% affected at baseline, 46% at 3 months and 29% at 6 months; for HS: 13% affected at baseline, 8% at 3 months and 7% at 6 months). No reduction in ppb counts were detected for MM in either group (Table 3).

**Table 3:** Patients affected by clinical noticeable halitosis vs. DMS, HS and MM status

			OPP affected	Total OPP	TAU affected	Total TAU	Total affected	Total	*p*-value: OPP vs. TAU
Dimethyl sulfide (threshold: 24 ppb)	Baseline	Count	10	15	8	15	18	30	0.710
		% within group	67		53		60		
	After 3 months	Count	6	13	8	13	14	26	0.695
		% within group	46		62		54		
	After 6 months	Count	4	14	9	15	13	29	0.139
		% within group	29		60		45		
Hydrogen sulfide (threshold: 95 ppb)	Baseline	Count	2	15	4	15	6	30	0.651
		% within group	13		27		20		
	After 3 months	Count	1	13	5	13	6	26	0.160
		% within group	8		39		23		
	After 6 months	Count	1	14	3	14	4	28	0.596
		% within group	7		21		14		
Methyl mercaptan (threshold: 12 ppb)	Baseline	Count	4	15	4	15	8	30	1.0
		% within group	27		27		27		
	After 3 months	Count	3	13	4	13	7	26	1.0
		% within group	23		31		27		
	After 6 months	Count	4	14	6	14	10	28	0.695
		% within group	29		43		36		

*p*-value according to Fisher’s Exact Test between OPP and TAU, significance at 5%. DMS, dimethyl sulfide; HS, hydrogen sulfide; MM, methyl mercaptan; OPP, oral preventive program; TAU, treatment as usual

### Winkel index

The mean Winkel Index scores within both groups were 5.5 (SD 2.2) at baseline, 3.3 (SD 2.5) after 3 months and 2.9 (SD 1.8) after 6 months.

**Fig. 3:**
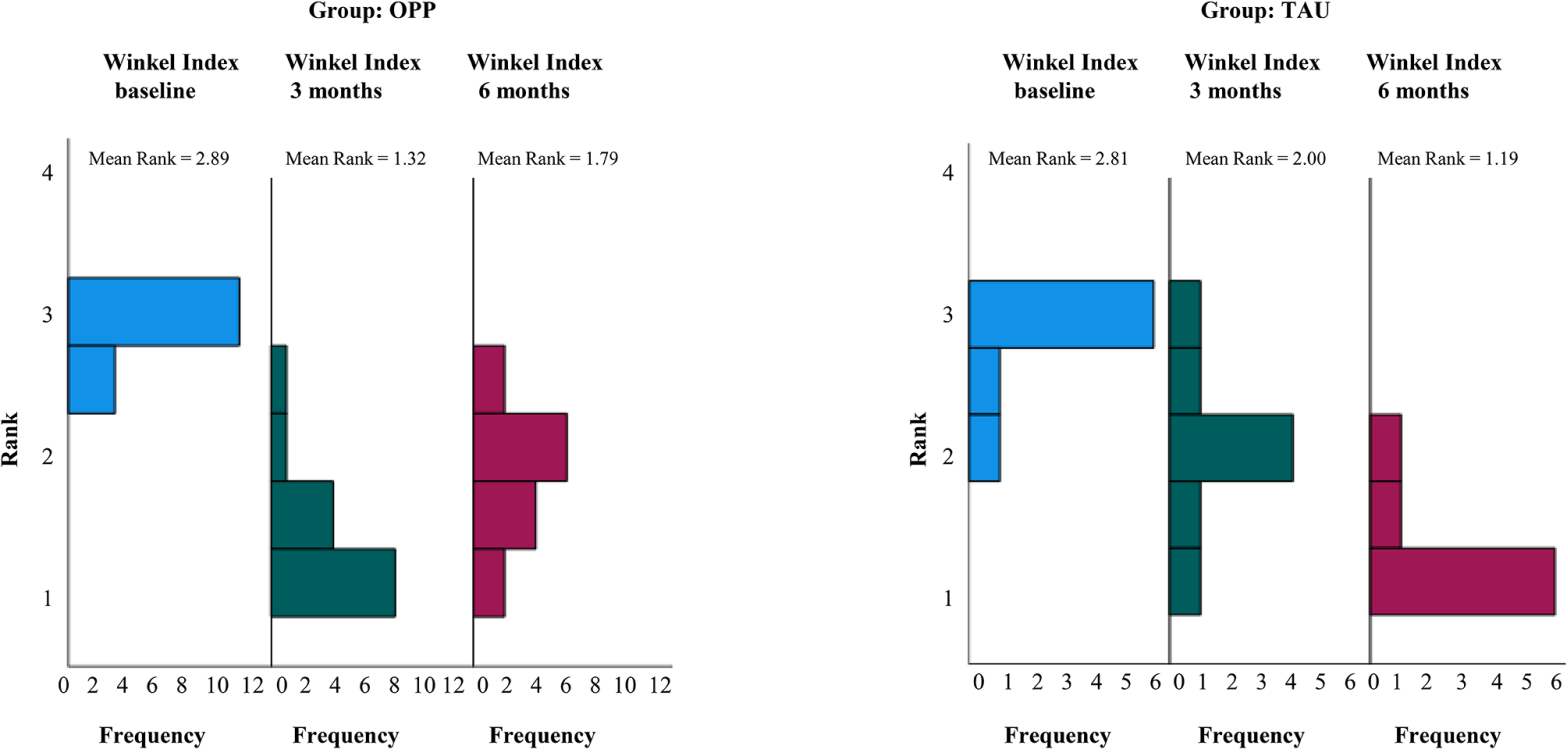
Winkel Index at baseline and after 3 and 6 months for both groups. *P*-value according to Friedman’s two-way analysis of variance by ranks, significance at 5%

When looking at potential differences between both groups, a significant reduction in the mean Winkel Index score was observed between baseline and 3 months after study start for the OPP group, and between baseline and six months after study start for the TAU group (Fig. 3).

At baseline, HS and MM levels correlated with Winkel Index scores (Pearson’s r 0.544, *p* = 0.003 and 0.553, *p* = 0.002, respectively), meaning that higher HS and MM levels were associated with higher Winkel Index scores; a strong correlation was also found between HS and MM (Pearson’s r 0.977, *p* < 0.001). Furthermore, HS levels correlated with Winkel Index scores after 3 months (Pearson’s r 0.425, *p* = 0.043). DMS showed no noticeable correlation with Winkel Index scores or the other two VSCs at any time point.

At baseline, Winkel Index scores correlated with mean PBI scores (Pearson’s r 0.395, *p* = 0.038). This correlation proved to be more profound 3 months after study start (Pearson’s r 0.676, *p* < 0.001). After 3 months; there was also a correlation between Winkel Index scores and mean QHI scores (Pearson’s r 0.797, *p* < 0.001). Six months after study start, QHI scores correlated with Winkel Index scores (Pearson’s r 0.648, *p* < 0.001).

### Patient- and proxy-reported halitosis and impairments

Reports from patients and mothers about perceived halitosis and its possible impairments differed greatly. None of the included CKD patients reported self-perceived halitosis, in contrast to most of their mothers (*n* = 25). Mothers reported bad breath at different points during the day, but were not able to differentiate specific malodors such as uremic breath. Some of the questioned mothers found that tooth brushing reduced halitosis in their children. None of the mothers who thought that their child suffered from halitosis reported psychosocial impairments due to bad breath.

*Yes, my son often smells unpleasantly out of his mouth. He seems to not notice it himself and when I recognize it, I mean, the bad breath, I often tell him to brush his teeth more frequently. He doesn’t want to hear this, of course, because he has so much to think and care about due to his illness. But healthy teeth and a pleasant breath are so important, too! On the other hand, I don’t have the feeling that he’s being isolated or else because of this smelling-issue by his friends. So, sometimes, I just let him be and don’t bother him even more; the poor kid.*

All of the nurses who were asked in the course of CKD unit visits found that halitosis was frequently present in young CKD patients in general, as well as in the included patients. All of them found that the bad breath possibly accounted for a reduced quality of life.

*These kids suffer from bad breath frequently. I am not sure where it comes from, but I think it’s way more often than in kids without CKD. I just think that the CKD and its therapy takes up their whole life and so they do not really bother about smelling out of their mouths. Then again, I sometimes have the feeling, that especially young girls are not happy with their bad breath and maybe, their shyness and seclusion results from this condition.*

One nurse talked about specific CKD patients who not only suffered from halitosis, but also from unpleasantly perspiring feet without noticing. Interestingly, another nurse reported, that in some patients, the longer their dialysis had been in the past, the more they suffered from halitosis.

## Discussion

According to this subanalysis of a randomized clinical trial, young CKD patients are affected by halitosis. In adult patients with CKD, halitosis is already a well-documented problem compared to healthy individuals [5], which apparently occurs in children and adolescents at the onset of the disease. In this secondary analysis of the present study, OralChroma measurements were focused on. Patients with CKD showed halitosis at baseline. Yet no differences in VSC levels were found between intervention and control group during the course of the study. Due to randomization, the initial mean VSC scores for HS and MM were unintentionally higher in the TAU group than in the OPP group. Table 2 demonstrates how few statistical outliers lead to such differences, which were not clinically relevant (Table 3). Among all VSCs, DMS seemed to be the main source of halitosis within the study participants. As high DMS levels are usually present in the context of extra-oral, blood-borne halitosis, it was not surprising that these ppb levels did not significantly improve after intense dental prophylaxis measures.

Yet there was a tendency towards a reduction in DMS and HS in affected patients with values representing clinically-noticeable oral malodor within the OPP group over time. When looking at potential differences between both groups with regard to tongue coating, significant differences were observed between baseline and 3 months after study start for the OPP group, and between baseline and 6 months after study start for the TAU group. Thus, an adequate oral hygiene prophylaxis program – as in the OPP group – seemed to contribute to decreased VSC levels. Although not tested within this study group, recent results of a meta-analysis by Szalai et al. showed that the additional use of chlorine dioxide mouthwashes play an important role in the supportive treatment of halitosis, especially for patients with elevated HS levels [21]. Additionally, and in line with previous research [22], results from the current study indicated high tongue coating levels at baseline that correlated significantly with high PBI and/or QHI scores [23].

Despite the existing gingivitis of the study patients, MM was not the main volatile component. Although the reduction in DMS was not significant, it appears reasonable to pursue dental prevention to reduce both gingivitis and oral gases. Successful prevention of gingivitis with a needs-related program can prevent or delay onset of periodontitis as another possible cause of VSC source, especially for this vulnerable group of patients. The typical “uremic fetor” is frequently associated with advanced CKD, mostly due to exhalation of ammonia, dimethylamine and trimethylamine [24]. As kidney dysfunctions lead to impaired removal of waste products from the blood, urea and creatinine (among others) are metabolized to ammonia. Excess ammonia, as well as nitrogen-containing volatile compounds like methylamines, can diffuse into the lungs and are shown in higher concentrations in the breath of end-stage CKD patients compared to healthy controls [25, 26]. Whereas in the vast majority of adult CKD patients, ageing and/or lifestyle-associated comorbidities have to be considered and may bias the results, these issues are less relevant in pediatric CKD patients [27].

Another focus of the present study was to investigate the self-perceived oral malodor in the course of an extended anamnesis of the included patients and their mothers at the dental clinic. Listening to patient perspectives and experiences, especially those of children, seems to be a highly necessary aspect of patient-centered care of chronic diseases [28]. While halitosis was regularly reported by mothers and confirmed by professionals (e.g. nurses), the patients themselves did not speak about potential halitosis and did not regard it as a problem. Missing self-perception could be a result of adopted social desirability in an effort to achieve social acceptance [29]. It is a known phenomenon that caregivers may rate their children’s quality of life lower than the children with CKD themselves, since caregivers may expect an uncertain and difficult future for these children. Children with CKD, on the other hand, may view their life situation more positively than their caregivers due to their temporal orientation to the present [30]. In addition, young CKD patients often suffer from multiple symptoms due to their illness, and are not able to differentiate or specify one particular healthcare problem like halitosis [28, 31–33]. Most often, the main caregivers are the mothers of affected children, as in the current study, and are very sensitive at detecting distress in their children [30].

This study has some limitations. Because this was a secondary analysis within this interdisciplinary randomized, controlled trial, the sample size calculation did not focus on halitosis or, in specific, VSC measurements. Yet, measurements (in ppb) are presumably even more precise and selective than clinical indices as PBI. Furthermore, the difficulty of controlling highly individual VSCs can be critically discussed. This was accounted for through instructing all study participants regarding food and oral hygiene prior to study measurements. Yet, it cannot be completely ruled out, that potentially hidden spice/herb additions to snacks or convenience foods might have still influenced VSCs to some extent. Additionally, it would have been beneficial to explore not only tongue coating and VSCs, but also organoleptic measurements as they have been described to be highly sensitive [34]. Since halitosis was a secondary endpoint we refrained from integrating even more study-related expenses for study participants, holding the fact, that young patients with CKD already undergo numerous medical examinations due to their disease.

The results of this subanalysis of a randomized clinical trial highlight the significance of halitosis in young CKD patients. It is highly important to deal with the type of disease and treatment in this vulnerable patient group. Furthermore, optimal daily oral hygiene and an adequate oral hygiene prophylaxis program might contribute to decreased VSC levels in young CKD patients, reducing the burden of the multifactorial medical condition of these patients.

## Conclusions

Children, adolescents and young adults with CKD regularly suffer from halitosis. The burden of increased halitosis is perceived by parents and staff of affected patients. Dimethyl sulfide is the main source of this extra-oral blood-borne halitosis resulting from CKD, which could not be significantly reduced in the present study, even after intense dental prophylaxis measures. The main effect of preventive measures resulted in a reduction of tongue coating and consequently in a tendency towards reduced hydrogen sulfide concentrations but methyl mercaptan concentrations were unaffected.

## Data Availability

The data underlying this article cannot be shared publicly for no clear reason due to privacy issues of individuals that participated in the study. Yet, data can be shared on reasonable request to the corresponding author.

## Abbreviations

CAKUT: Congenital anomalies of the kidney and urinary tract
CKD: Chronic kidney disease
DMS: Dimethyl sulfide
HS: Hydrogen sulfide
MM: Methyl mercaptan
OPP: Oral preventative program
PBI: Papillary Bleeding Index
QHI: Quigley Hein Index
SD: Standard deviation
TAU: Treatment as usual
VSC: Volatile sulfur compound

## References

[1] Silva MF, Leite FRM, Ferreira LB, Pola NM, Scannapieco FA, Demarco FF (2018). Estimated prevalence of halitosis: a systematic review and meta-regression analysis. Clin Oral Investig.

[2] Memon MA, Memon HA, Muhammad FE, Fahad S, Siddiqui A, Lee KY et al. Aetiology and associations of halitosis: a systematic review. Oral Dis. 2022. 10.1111/odi.14172 35212093

[3] Guedes CC, Bussadori SK, Weber R, Motta LJ, Costa da Mota AC, Amancio OMS (2019). Halitosis: prevalence and association with oral etiological factors in children and adolescents. J Breath Res.

[4] AlMadhi NA, Sulimany AM, Alzoman HA, Bawazir OA. Halitosis in Children Undergoing Full Mouth Rehabilitation under General Anesthesia. Child (Basel). 2021;8(2). 10.3390/children8020149 PMC7922509 33671154

[5] Santaella NG, Simpione G, Maciel AP, Lauris JR, da Silva Santos PS (2021). Volatile sulphur compounds in people with chronic kidney disease and the impact on quality of life. Odontology.

[6] Ortiz V, Filippi A, Halitosis (2021). Monogr Oral Sci.

[7] Tangerman A. Halitosis in medicine: a review. Int Dent J. 2002;52 Suppl 3:201 – 16. 10.1002/j.1875-595x.2002.tb00925.x 12090453

[8] Harvey-Woodworth CN (2013). Dimethylsulphidemia: the significance of dimethyl sulphide in extra-oral, blood borne halitosis. Br Dent J.

[9] Richter JL. Diagnosis and treatment of halitosis. Compendium Continuing Educ Dentistry (Jamesburg NJ: 1995). 1996;17(4):370–24. quiz 88. 9051972

[10] Conceicao MDD, Giudice FS, Carvalho LF (2018). The Halitosis consequences Inventory: psychometric properties and relationship with social anxiety disorder. BDJ Open.

[11] Briceag R, Caraiane A, Raftu G, Horhat RM, Bogdan I, Fericean RM et al. Emotional and Social Impact of Halitosis on adolescents and young adults: a systematic review. Med (Kaunas). 2023;59(3). 10.3390/medicina59030564 PMC10057342 36984565

[12] Zaitsu T, Ueno M, Shinada K, Wright FA, Kawaguchi Y (2011). Social anxiety disorder in genuine halitosis patients. Health Qual Life Outcomes.

[13] Deutscher H, Derman S, Barbe AG, Seemann R, Noack MJ (2018). The effect of professional tooth cleaning or non-surgical periodontal therapy on oral halitosis in patients with periodontal diseases. A systematic review. Int J Dent Hyg.

[14] AlMadhi NA, Sulimany AM, Alzoman HA, Bawazir OA (2021). Halitosis and Associated Risk factors in children: a cross-sectional study. J Contemp Dent Pract.

[15] Winkel EG, Roldan S, Van Winkelhoff AJ, Herrera D, Sanz M (2003). Clinical effects of a new mouthrinse containing chlorhexidine, cetylpyridinium chloride and zinc-lactate on oral halitosis. A dual-center, double-blind placebo-controlled study. J Clin Periodontol.

[16] Mann JFE, Chang TI, Cushman WC, Furth SL, Ix JH, Hou FF (2021). Commentary on the KDIGO 2021 Clinical Practice Guideline for the management of blood pressure in CKD. Curr Cardiol Rep.

[17] Tangerman A, Winkel EG (2008). The portable gas chromatograph OralChroma™: a method of choice to detect oral and extra-oral halitosis. J Breath Res.

[18] Tangerman A, Winkel EG (2010). Extra-oral halitosis: an overview. J Breath Res.

[19] Mayring P. Qualitative Inhaltsanalyse. Grundlagen und Techniken 2010.

[20] Axelsson P, Lindhe J (1974). The effect of a preventive programme on dental plaque, gingivitis and caries in schoolchildren. Results after one and two years. J Clin Periodontol.

[21] Szalai E, Tajti P, Szabó B, Hegyi P, Czumbel LM, Shojazadeh S (2023). Daily use of chlorine dioxide effectively treats halitosis: a meta-analysis of randomised controlled trials. PLoS ONE.

[22] Choi HN, Cho YS, Koo JW. The Effect of Mechanical Tongue cleaning on oral Malodor and Tongue Coating. Int J Environ Res Public Health. 2021;19(1). 10.3390/ijerph19010108 PMC8751028 35010368

[23] Seemann R, Conceicao MD, Filippi A, Greenman J, Lenton P, Nachnani S (2014). Halitosis management by the general dental practitioner–results of an international consensus workshop. J Breath Res.

[24] Simenhoff ML, Burke JF, Saukkonen JJ, Ordinario AT, Doty R (1977). Biochemical profile or uremic breath. N Engl J Med.

[25] Schönermarck U, Dengler C, Gmeinwieser A, Praun S, Schelling G, Fischereder M (2016). Exhaled breath volatile organic and inorganic compound composition in end-stage renal disease. Clin Nephrol.

[26] Neri G, Lacquaniti A, Rizzo G, Donato N, Latino M, Buemi M (2012). Real-time monitoring of breath ammonia during haemodialysis: use of ion mobility spectrometry (IMS) and cavity ring-down spectroscopy (CRDS) techniques. Nephrology, dialysis, transplantation: official publication of the European Dialysis and Transplant Association. Eur Ren Association.

[27] Obermeier J, Trefz P, Happ J, Schubert JK, Staude H, Fischer DC (2017). Exhaled volatile substances mirror clinical conditions in pediatric chronic kidney disease. PLoS ONE.

[28] Schalkers I, Parsons CS, Bunders JF, Dedding C (2016). Health professionals’ perspectives on children’s and young people’s participation in health care: a qualitative multihospital study. J Clin Nurs.

[29] Bailey PK, Hamilton AJ, Clissold RL, Inward CD, Caskey FJ, Ben-Shlomo Y (2018). Young adults’ perspectives on living with kidney failure: a systematic review and thematic synthesis of qualitative studies. BMJ Open.

[30] Kiliś-Pstrusińska K, Medyńska A, Chmielewska IB, Grenda R, Kluska-Jóźwiak A, Leszczyńska B (2013). Perception of health-related quality of life in children with chronic kidney disease by the patients and their caregivers: multicentre national study results. Qual Life Res.

[31] Wijngaarde RO, Hein I, Daams J, Van Goudoever JB, Ubbink DT (2021). Chronically ill children’s participation and health outcomes in shared decision-making: a scoping review. Eur J Pediatr.

[32] Gutman T, Hanson CS, Bernays S, Craig JC, Sinha A, Dart A (2018). Child and parental perspectives on communication and decision making in Pediatric CKD: a Focus Group Study. Am J Kidney Dis.

[33] Lambert V, Glacken M, McCarron M (2013). Meeting the information needs of children in hospital. J Child Health Care.

[34] Szalai E, Tajti P, Szabó B, Kói T, Hegyi P, Czumbel LM (2023). Organoleptic and halitometric assessments do not correlate well in intra-oral halitosis: a systematic review and meta-analysis. J Evid Based Dent Pract.

